# Progress in Medicine

**Published:** 1894-09-29

**Authors:** 


### Progress in Medicine.

### DISEASES OF THE HEART AND VASCULAR SYSTEM.

**The Venous Pulse.**—Dr. James Mackenzie[11] has afforded most valuable information on the significance of the venous pulse by means of cardiographic and sphygmographic tracings, recording simultaneously the carotid and venous pulse in the neck and also in the liver in certain cases. The tendency of the right heart to dilate and to permit a regurgitant stream of blood to flow back into the auricle during systole of the ventricle when undue opposition is offered to the performance of its work, has long been recognised, and the manifestation of this regurgitation is chiefly in the veins at the root of the neck. Mackenzie has been able to prove that the pulse in the veins of the neck is not always a ventricular pulse, in fact, three distinct sources of pulsation are recognisable, (1) auricular, (2) ventricular, (3) arterial, communicated from the adjacent carotid. While the possible presence of the latter should be borne in mind, it is without significance, and that it is really due to a pulsation communicated from the carotid is shown, first, by its absence in a case in which the carotid pulse was absent; secondly, by its being always synchronous with the carotid pulse; thirdly, by its invariable absence in situations where no large artery is adjacent, *e.g.*, the liver. The true venous pulse may be divided into two forms, viz.: (*a*) the auricular venous pulse, and (*b*) the ventricular venous pulse.

The first is that form which presents distinct evidence of the functional activity of the right auricle. In this form there is also a wave due to the ventricle, and as it increases the auricular wave decreases, and finally disappears, and thus the form called the ventricular venous pulse is developed. The ventricular venous pulse is but a more advanced stage than the auricular; for during its development there is a gradual fading of the auricular wave, there is a period when such terms do not sufficiently denote the character of the pulse. But the terms are convenient for descriptive purposes. The ventricular venous pulse only appears when there is organic disease of the heart itself (most commonly in valvular disease). When the failure of the heart is functional, and not due to organic disease of the valves, the auricular pulse persists to the end.

One of the most interesting points brought out by Dr. Mackenzie's most valuable paper, is the fact that we can thereby obtain some measure of the degree of incompetence of the tricuspid valve. Thus the time of appearance of the pulse due to the ventricular systole is regulated by the time it appears after the auricular pulse. As this wave is due to blood regurgitating through the tricuspid orifice during the ventricular systole, and as during the ventricular systole the auricle is in diastole and is sucking blood into its cavity, the wave cannot appear in the veins till the auricle is filled. The over-plus sent back from the right ventricle then appears as a wave in the veins of the neck. Usually the wave due to the ventricular systole is divided into two parts by a notch of varying depth. The bottom of the notch indicates the time of the closure of the pulmonary valves, the part preceding the notch being due to the ventricular systole after the auricle has been distended. The greater the tricuspid incompetence the sooner will the auricle be filled, and the earlier will this portion of the ventricular wave appear.

Similar types of pulses may be recognised in the liver. Here the pulse only appears when there is organic disease of the heart.

The observation of the venous pulse serves, with the left heart action, as the best means of determining the lack of harmony between the contraction of the different heart chambers. The cases of irregular heart action studied by Dr. Mackenzie are divided into seven groups, viz.: (1) In which there was complete agreement in relative rhythm during certain phases of irregularity of the right auricle and ventricle and left ventricle; (2) in which there was an exaggerated auricular impulse during this period of irregularity; (3) in which there was maintenance of the periodicity of action of the right auricle during irregular action of both ventricles; (4) in which there was persistence of the auricular systole during a pause in the ventricular systole; (5) in which there was contraction of the right ventricle during a pause of the left; (6) in which there was complete discordance in the action of the two sides of the heart; (7) in which there was a probable displacement of the relative time of the right and left heart movements.

**Pericardial Effusion, Signs of Commencing.**—Contrary to the usually accepted view that commencing pericarditis is first manifested by an increase (broadening) of the cardiac dulness of the base of the heart, Ebstein[12] finds that the first alteration occurs in the lower regions of the area of cardiac dulness. First there is stretching of the pericardium to the left side (seldom discoverable, partly on account of the simultaneous occurrence of left pleurisy, and partly on account of the loud tympanitic note in the semilunar space). Secondly there is distension of the pericardial sac to the right side, and this enlargement may be recognised clinically in nearly every case by the appearance of an absolute, or almost absolute, dulness on the fifth right intercostal space, in the region named by Ebstein the cardio-hepatic angle.

**Tumours of the Heart.**—Pavlowski[13] records clinical observations in a case of polypoid tumour of the left auricle, but comes to the conclusion that it is impossible to make a diagnosis during life.

**Wounds of the Pericardium**—Amerio,[14] *apropos* of a case occurring in his practice, made some experiments and arrived at the following conclusions as the result: (1) Animals may live in apparent health, even after wounds which have resulted in complete pericardial adhesion. (2) Pericardial surgery should be further developed, so as to render certain the prevention of septic infection by wounds of neighbouring organs. (3) In cases in which it is certain that the pericardium has been wounded, especially if the lesion has been made with septic instruments, surgical intervention is justifiable. It should consist in enlarging the pericardial wound, washing out all blood and pus from the sac, and thoroughly disinfecting it. (4) In certain cases, in which thoracic tumours have invaded the pericardium, the affected portion of the sac may be removed without prejudicing the patient's life.

**Adherent Pericardium.**—The observations of Dr. Theodore Fisher[16] point to the conclusion that pericarditis, with adherent pericardium, is a very fatal complication, at least in children. Adherent pericardium is the most common cause of a dilated heart in childhood, and death from such a cause is far more common than from mitral stenosis. Thus the post-mortem reports at Guy's for seven years showed that there were only three deaths from mitral stenosis of the age of fifteen years and under, while there were thirteen deaths from cardiac disease with adherent pericardium in which valvular disease was either absent or there was merely slight thickening of the mitral valves. Apparently very few children with adherent pericardium survive the age of fifteen, while mitral stenosis is not uncommon in young adults. This suggests that the immediate prognosis is considerably worse in the former affection.

**Diastolic Bruit at the Apex of the Heart.**—Dr. Fisher[17] adduces most interesting and instructive facts, and quotes various authorities, showing that a presystolic bruit in children, most audible at the apex, may be present without aortic disease and with a dilated mitral orifice. As this is a physical symptom not generally recognised, his contribution is worthy of careful note.

**Ulcerative Septic Endocarditis.**—Drs. Widal and Bezancon[17] obtained some interesting results from inoculation of rabbits in the subcutaneous tissues of the ear with streptococci. So long as streptococci from the mouth in a normal condition, and therefore not virulent, were inoculated no result was obtained. But by reinforcing the virulence of the streptococci with a variety of bacillus coli the inoculation gave rise to a patch of erysipelas, and a streptococcus derived from this patch, inoculated in a rabbit, set up first an erysipelas, and then a vegetative endocarditis, in all respects similar to the same affection in man. There was no previous traumatism affecting the valves, nor intravenous injection, it being the intention of the experimenters to reproduce as nearly as possible the clinical conditions in man.

Another interesting pathological fact comes from the bulletin of the Johns Hopkins Hospital, W. T. Howard, junr., reporting a case of ulcerative endocarditis, showing the bacillus diphtheriæ, and this is the first observation of this bacillus as an etiological factor in acute ulcerative endocarditis, affording as it does an explanation of the occurrence of endocarditis as a complication of diphtheria.

Mr. John Burgess[18] records an interesting case in a lady in whom primary septic endocarditis occurred with no obvious cause, it merely following on a chill.

The *diagnosis* of septic endocarditis is sometimes very difficult, and Dr. Sansom[19] has stated that "the continued manifestation of very low tension, with murmurs, perhaps, of very slight intensity, there being marked physical depression and, perhaps, some slight mental disturbance, justify the diagnosis of grave endocarditis of septic origin. It has been thought that the diagnosis of septic endocarditis may be made from an inspection of the temperature chart—that the peaks representing high elevations and rapid falls are characteristic of the disease—but I have found that this sign cannot be relied upon, for the grave disease can progress without elevation of temperature. It is in cases where the diagnosis is very difficult that the observation of the vaso-motor paralysis, indicated by the extremely dicrotic pulse, comes as an important indication."

[11] Edin. Med. Jour., June, 1894; Jour. of Path. and Bac., vol. ii., Nos. 1 and 3. [12] Visch. Archiv., vol. cxxx., No. 3, to Arch. f. Klin. Med., No. 37, 1893; Pract., February, 1894. [13] Bolnitschnaja Gaz., Botkina, 1893; cent. f.d. Med., Wissensch, Dec., 1893; Internat. Med. Mag., May, 1894, p. 308. [14] Med. Rec., May 19, 1894, p. 638. [15] Brit. Med. Jour., April 28, 1894. [16] Ibid. [17] Sc. Med. des Hop., April 20; Med. Week., April 27, 1894. [18] Dub. Jour. of Med. Sc., April, 1894, p. 308. [19] Brit. Med. Jour, April 14, 1894.

